# Psychological distress, resilience, and well-being among survivors of the 2023 Kahramanmaraş earthquakes: a multi-site cross-sectional study

**DOI:** 10.3389/fpsyg.2025.1730083

**Published:** 2025-12-10

**Authors:** Eda Yılmazer

**Affiliations:** Department of Psychology, Faculty of Social Science, Beykoz University, Istanbul, Türkiye

**Keywords:** Kahramanmaraş earthquakes, post-traumatic stress disorder, depression, anxiety, resilience, well-being

## Abstract

**Background:**

The 2023 Kahramanmaraş earthquakes were among the most devastating disasters in modern Türkiye, with severe consequences for survivors’ mental health. While post-disaster psychopathology has been widely studied, less is known about positive psychological outcomes such as resilience and well-being in this context.

**Objective:**

This study examined levels of depression, anxiety, post-traumatic stress symptoms, well-being, and resilience among earthquake survivors, explored group differences, and identified predictors of key psychological outcomes.

**Methods:**

A cross-sectional survey was conducted with 642 adult survivors across 11 heavily affected cities, nearly 2 years after the earthquakes. Data were collected between September 2024 and January 2025, corresponding to 19–22 months after the earthquakes. Participants completed validated self-report measures: BDI-II, BAI, PCL-5, WEMWBS, and CD-RISC. Analyses included group comparisons, correlations, hierarchical regressions predicting well-being and resilience, and logistic regression predicting probable PTSD (PCL-5 ≥ 47).

**Results:**

Survivors reported moderate depression (*M* = 22.1), mild–moderate anxiety (*M* = 19.4), and near-threshold PTSD symptoms (*M* = 40.0). Overall, 31.0% of participants exceeded the PCL-5 cut-off for probable PTSD (95% CI: 27.4–34.8%). Well-being (*M* = 35.6) and resilience (*M* = 56.0) were below normative values. Women reported higher depression than men, while trauma-exposed participants had significantly worse outcomes across all measures. Bivariate correlations showed strong associations between distress, reduced well-being, and diminished resilience. Hierarchical regressions indicated that depression and PTSD symptoms were the primary predictors of both lower well-being (*R*^2^ = 0.45) and resilience (*R*^2^ = 0.32). Logistic regression revealed that female gender (OR = 1.80, *p* = 0.024) and depression severity (OR = 1.10, *p* = 0.001) significantly increased the likelihood of probable PTSD.

**Conclusion:**

Nearly 2 years after the earthquakes, survivors experienced substantial psychological distress alongside reduced well-being and resilience. Clinical symptoms, particularly depression and PTSD, more strongly associated with outcomes than sociodemographic or exposure variables. These findings highlight the need for interventions that both alleviate trauma-related psychopathology and strengthen resilience and well-being as part of long-term disaster recovery.

## Introduction

The twin earthquakes that struck southeastern Türkiye on 6 February 2023 represent one of the most devastating natural disasters in recent history, causing extensive destruction across 11 provinces. With moment magnitudes of 7.7 and 7.6, the events leveled large sections of urban areas in Kahramanmaraş, Hatay, Gaziantep, Adıyaman, Malatya, and surrounding regions, resulting in more than 50,000 fatalities and the displacement of millions of residents (Şenol Balaban et al., 2025). Beyond the severe physical damage, disasters of this scale are consistently associated with heightened risks for a broad range of psychological difficulties (Heanoy and Brown, 2024; Şam et al., 2025; Satıcı et al., 2024; Balbay et al., 2024), particularly post-traumatic stress disorder (PTSD) (Sirotich and Camisasca, 2024), depression, and anxiety (Güler et al., 2024) among survivors. Epidemiological evidence indicates that nearly one-third of disaster-exposed individuals may experience clinically significant psychopathology—such as PTSD, depressive disorders, or anxiety disorders—during the post-disaster period (Fong et al., 2022; Hosseinnejad et al., 2022; Tahernejad et al., 2023). Earthquakes, due to their sudden onset and life-threatening nature, appear to be especially potent in this regard: a meta-analysis of 46 studies reported that approximately 23–30% of earthquake survivors meet criteria for PTSD within the first post-disaster year, with even higher rates observed in severely affected communities (Dai et al., 2016).

Research on previous large-scale earthquakes consistently identifies several groups and characteristics associated with heightened psychological vulnerability. Women, for example, frequently report higher levels of post-disaster psychological morbidity than men, a pattern that has been documented across diverse cultural and disaster contexts (McKinzie and Clay-Warner, 2021). Elevated trauma exposure—such as being trapped under collapsed structures, sustaining physical injuries, losing close relatives, or witnessing severe destruction—is also strongly associated with greater post-disaster mental health difficulties (Schwind et al., 2019). Socioeconomic and demographic factors, including low income, limited educational attainment, and in some studies older age, further correspond to increased risk for PTSD and depression (Mao and Agyapong, 2021; Çınaroğlu et al., 2025),. While the earlier study in which I contributed as a second author examined the psychological effects of the 2023 earthquakes among indirectly exposed non-victims, the present investigation is distinct in directly assessing survivors residing in 11 severely affected cities nearly 2 years after the disaster. Conversely, robust social support and adaptive coping capacities appear to buffer individuals against persistent trauma-related symptoms (Can Gür, 2024). Collectively, these observations are consistent with the broader disaster mental health literature, which highlights a dose–response pattern between trauma severity and psychological distress and underscores the protective role of psychosocial resources in fostering resilience.

The 2023 Kahramanmaraş earthquakes provide a critical context for examining the psychological consequences of large-scale natural disasters. Given the unprecedented magnitude of this event in modern Türkiye, there remains an urgent need to document its mental-health impact and to identify factors that may mitigate or exacerbate survivors’ psychological outcomes. Early reports have already indicated widespread distress among affected populations (Çınaroğlu et al., 2025), including previous work in which I contributed as a second author. That prior study synthesized existing evidence through a systematic review and meta-analysis, whereas the present investigation contributes original, large-scale primary data collected directly from survivors nearly 2 years after the disaster. Empirical findings from the region support the severity of the psychosocial burden: for example, a cross-sectional survey conducted in a severely affected district (Nurdağı, Gaziantep) found that more than 51% of adult survivors screened positive for psychological distress in the months following the earthquakes, with particularly elevated risk among women, injured individuals, and those who had lost first-degree relatives (Tomak et al., 2024). Additional early assessments from the affected provinces similarly reported high rates of probable PTSD, with some estimates suggesting that over half of adult survivors met screening thresholds within the first post-disaster year (Yakşi and Eroğlu, 2024; Taşkın et al., 2025; Tanrikulu and Kayaoglu, 2024). Key earthquake-related stressors—such as persistent aftershock anxiety, bereavement, extensive property destruction, and the loss of one’s broader community environment—have been identified as major contributors to these psychological reactions. While these findings underscore the profound and widespread mental health burden created by the 2023 earthquakes, they also highlight the heterogeneity of survivors’ experiences; not all individuals are affected to the same degree. Identifying the predictors of heightened vulnerability, and understanding the mechanisms through which risk accumulates, is therefore essential for developing targeted and effective post-disaster interventions (Ural et al., 2024).

Beyond the prevention and treatment of psychiatric disorders, there is increasing recognition of the importance of examining positive mental health constructs in disaster research. Traditional post-disaster studies have largely emphasized trauma-related symptoms—such as PTSD, depression, and anxiety—while comparatively underrepresenting survivors’ resilience and overall well-being. Yet resilience, broadly conceptualized as the capacity to adapt, recover, or grow in the face of adversity, is now understood to be a critical component of post-disaster adjustment (Oliva and Lazzeretti, 2019; Zhao et al., 2020). Higher levels of psychological resilience have been associated with reduced likelihood of developing PTSD and other mental health difficulties following traumatic events (Snijders et al., 2018). Empirical evidence from prior earthquake contexts similarly indicates that individuals possessing stronger resilience resources—such as effective coping strategies, robust social support, and dispositional optimism—tend to report fewer PTSD symptoms and demonstrate more rapid psychological recovery (Xi et al., 2020; Chen et al., 2022; Ikizer et al., 2016).

In recent years, theoretical models in mental health have emphasized the importance of evaluating both negative and positive indicators of psychological functioning following major traumatic events. The dual-continuum model of mental health posits that psychological distress (e.g., PTSD, depression, anxiety) and psychological well-being operate on related but distinct continua; thus, the presence of distress does not automatically preclude well-being, and improvements in one domain do not necessarily guarantee changes in the other. Complementing this view, contemporary resilience theory conceptualizes resilience as a dynamic system of psychological and social resources—such as coping self-efficacy, emotional regulation, and meaning-making—that buffer the adverse effects of trauma exposure and support adaptive functioning over time. From a broader ecological perspective, disaster mental health frameworks (e.g., Norris et al., 2008) propose that post-disaster adjustment reflects an interplay between risk factors (e.g., trauma severity, bereavement, demographic vulnerabilities) and protective resources (e.g., resilience, coping capacities, social support), with both losses and gains in these resources shaping survivors’ psychological trajectories. Together, these theoretical perspectives provide the conceptual foundation for the present study by framing psychological distress, resilience, and well-being as interconnected yet distinct components of post-disaster functioning, and by highlighting the importance of assessing them simultaneously to capture the full psychological impact of the 2023 Kahramanmaraş earthquakes.

In parallel, mental well-being—encompassing domains such as life satisfaction, positive affect, and a sense of meaning or quality of life—represents an important outcome in its own right (Arguvanlı Çoban and Gün Koşar, 2025). Disasters can significantly erode subjective well-being; declines in life satisfaction and overall quality of life have been documented across multiple earthquake-affected populations. Following the 2023 Kahramanmaraş earthquakes, many survivors continue to report diminished well-being nearly 2 years after the event, particularly in emotional and social domains, with women and older adults often exhibiting greater vulnerability (Samanci Tekin and Aydin, 2024; Yanardağ et al., 2025),. Evaluating well-being alongside psychological distress therefore provides a more holistic understanding of the disaster’s impact and aligns with the World Health Organization’s definition of mental health as encompassing not merely the absence of illness but also the presence of positive psychological functioning (World Health Organization, 2022).

A growing body of disaster mental-health research emphasizes that psychological distress, resilience, and well-being form an interconnected system that jointly shapes survivors’ post-traumatic adjustment. Psychological distress—including PTSD symptoms, depression, and anxiety—reflects the emotional and physiological burden imposed by traumatic exposure, and elevated levels of distress are consistently linked to impaired functioning, reduced life satisfaction, and greater vulnerability to chronic psychopathology. In contrast, resilience represents a constellation of adaptive psychological resources, such as effective coping strategies, emotional regulation, optimism, and perceived competence, that enable individuals to withstand or recover from adversity. Resilience is understood not merely as the absence of distress but as an active, dynamic process that can buffer survivors against the negative consequences of trauma. Well-being, encompassing positive affect, life satisfaction, and psychological vitality, constitutes another distinct dimension of mental health: according to the dual-continuum model, well-being can vary independently of symptom severity and offers a broader lens through which to understand survivors’ functioning in the aftermath of disaster. Empirical studies have repeatedly shown that individuals with higher resilience tend to report lower levels of PTSD, depression, and anxiety, while simultaneously exhibiting greater well-being. Conversely, elevated distress is associated with both diminished resilience and lower subjective well-being, suggesting that distress can erode the psychological resources necessary for positive adaptation. Taken together, this literature highlights the importance of examining distress, resilience, and well-being not as isolated constructs but as mutually influencing dimensions of post-disaster mental health—an approach that is particularly relevant in large-scale traumatic events such as the 2023 Kahramanmaraş earthquakes.

Building on this background, the present study was designed to address these knowledge gaps through a systematic evaluation of earthquake-related psychological outcomes nearly 2 years after the 2023 Kahramanmaraş disaster. The study pursued three primary objectives: (1) to quantify levels of psychological distress—including PTSD symptoms, anxiety, and depression—as well as positive mental health indicators such as well-being and resilience among adult survivors; (2) to examine subgroup differences in these outcomes based on demographic characteristics and degree of trauma exposure; and (3) to identify variables associated with key psychological indicators, particularly PTSD symptom severity, overall mental well-being, and resilience.

By delineating these patterns, the study aims to support the development of targeted mental health interventions and to enhance understanding of the psychological processes that influence adaptation in the aftermath of a large-scale disaster. More broadly, the findings seek to address a significant gap in the current literature by situating the psychological impact of the 2023 earthquakes within a comprehensive framework that emphasizes not only the mitigation of trauma-related symptoms but also the promotion of resilience and well-being as integral components of long-term recovery.

### Study design

This study employed a multi-center, cross-sectional analytic design to investigate the psychological consequences of the 2023 Kahramanmaraş earthquakes. Data were collected from 11 severely affected cities in Türkiye, yielding a final sample of 642 adults. The research aimed to comprehensively assess depressive symptoms, anxiety, post-traumatic stress symptoms, mental well-being, and resilience among survivors, and to examine these outcomes in relation to a wide range of sociodemographic and earthquake-related variables. In addition to descriptive and comparative analyses, correlational tests were conducted to evaluate associations among psychological constructs, and multivariate models were used to identify variables linked with resilience, well-being, and probable PTSD caseness. Data collection followed a standardized protocol across all sites. Participants completed a Sociodemographic Information Form and a battery of validated self-report instruments, including the Beck Depression Inventory-II (BDI-II), Beck Anxiety Inventory (BAI), PTSD Checklist for DSM-5 (PCL-5), Warwick–Edinburgh Mental Well-Being Scale (WEMWBS), and Connor–Davidson Resilience Scale (CD-RISC). The study was designed and reported in accordance with the Strengthening the Reporting of Observational Studies in Epidemiology (STROBE) guidelines for cross-sectional research.

### Setting and participants

Data collection was conducted across 11 severely impacted cities over a three-month period between September 2024 and January 2025, corresponding to approximately 19–22 months after the earthquakes. This timing was intentionally selected to capture not only the acute psychological responses to the disaster but also the persistence of distress and resilience nearly 2 years post-event, thereby providing insight into longer-term patterns of adjustment. The affected cities varied considerably in the extent of destruction, displacement, and availability of recovery resources; however, clustering by city was not modeled, an issue later acknowledged as a limitation.

The research team consisted of the principal investigator and four master’s-level clinical psychology students, all residing in the affected regions and familiar with the local context. The author additionally visited field sites to supervise procedures and support standardized data collection. Participants were recruited through a multi-modal convenience sampling strategy, including direct outreach in temporary shelters, community centers, and local organizations, as well as recruitment via personal networks, WhatsApp groups, social media platforms, and community contacts. This approach enabled inclusion of both displaced individuals and residents living in the broader community.

A total of 753 individuals were initially approached. After applying eligibility criteria and excluding incomplete or invalid responses, the final analytic sample consisted of 642 adults. Inclusion criteria required participants to be at least 18 years old, to have resided in one of the earthquake-affected cities at the time of the disaster, and to be free of current psychiatric diagnoses, psychiatric medication use, or ongoing psychotherapy. These criteria were implemented to estimate symptom levels among survivors not currently receiving mental health treatment; however, this approach likely underestimates the broader post-disaster psychological burden and is addressed in the Limitations section. Individuals with cognitive impairments or other conditions impairing questionnaire completion were also excluded.

### Measures

#### Sociodemographic information form

Sociodemographic data were collected using a structured form that included age, gender, education level, marital status, employment status, household income, and earthquake-related experiences. Earthquake exposure was assessed through a set of dichotomous indicators capturing whether participants had lost family members, sustained injuries, or experienced displacement as a direct result of the disaster. The study did not incorporate a graded or standardized exposure-severity index capable of quantifying the extent of financial loss, degree of property damage, duration of displacement, or other humanitarian and social consequences. Accordingly, exposure was operationalized in a broad binary format (direct loss vs. no major loss), which supports subgroup comparisons but does not capture the full variability or multidimensional nature of earthquake-related adversity.

#### Beck Depression Inventory-II (BDI-II)

Depressive symptoms were assessed using the Beck Depression Inventory–II (BDI-II), a widely utilized self-report instrument developed as a revision of the original Beck Depression Inventory (Beck et al., 1996). The BDI-II consists of 21 items aligned with DSM-IV diagnostic criteria for depressive disorders, with each item rated on a 4-point scale (0–3), yielding total scores ranging from 0 to 63. Factor-analytic research generally supports a two-factor structure encompassing cognitive–affective and somatic–performance components. The Turkish adaptation of the instrument was validated by Kapci et al. (2008) in a sample comprising 362 nonclinical adults and 176 clinical outpatients diagnosed with depressive disorders. The scale demonstrated excellent internal consistency (Cronbach’s *α* = 0.90 in the nonclinical group and 0.89 in the clinical group), high test–retest reliability (r = 0.94), and strong convergent and discriminant validity. Cut-off scores established for the Turkish population are consistent with the original version: 0–12 indicates minimal depressive symptoms, 13–18 mild, 19–28 moderate, and 29–63 severe depression.

#### Beck Anxiety Inventory (BAI)

Anxiety symptoms were measured using the Beck Anxiety Inventory (BAI), a 21-item self-report instrument developed by Beck et al. (1993) to assess the severity of anxiety experienced during the preceding week. Each item is scored on a 4-point Likert scale ranging from 0 (“not at all”) to 3 (“severely”), yielding total scores between 0 and 63. Factor-analytic studies typically identify two principal dimensions: somatic anxiety and subjective anxiety. The Turkish adaptation of the BAI was validated by Ulusoy et al. (1998) in clinical and nonclinical samples, demonstrating excellent internal consistency (Cronbach’s *α* = 0.93), acceptable test–retest reliability (*r* = 0.75), and moderate correlations with depressive symptom measures, supporting discriminant validity. Exploratory factor analysis confirmed the two-factor structure, and the scale has been shown to effectively distinguish individuals with clinically significant anxiety from other diagnostic groups.

#### PTSD Checklist for DSM-5 (PCL-5)

Post-traumatic stress symptoms were assessed using the PTSD Checklist for DSM-5 (PCL-5), a 20-item self-report instrument developed by Weathers et al. (2018) in accordance with the DSM-5 diagnostic framework. Items correspond to the four core PTSD symptom clusters—re-experiencing, avoidance, negative alterations in cognition and mood, and hyperarousal—and are rated on a 5-point Likert scale ranging from 0 (“not at all”) to 4 (“extremely”), producing total scores between 0 and 80. The Turkish validation of the PCL-5 was conducted by Boysan et al. (2017) in a mixed sample of psychiatric outpatients and community participants. The scale demonstrated excellent internal consistency (*α* = 0.94 for the total score), with subscale reliabilities ranging from 0.78 to 0.87, and test–retest reliability coefficients between 0.64 and 0.78 over a two-week interval. Convergent validity was supported through strong correlations with measures of trauma-related symptoms, depression, and anxiety. For Turkish populations, a cut-off score of 47 has been recommended for identifying probable PTSD, yielding a sensitivity of 0.76 and a specificity of 0.69.

#### Warwick-Edinburgh Mental Well-Being Scale (WEMWBS)

Mental well-being was measured using the Warwick–Edinburgh Mental Well-Being Scale (WEMWBS), originally developed by Tennant et al. (2007) to assess hedonic and eudaimonic components of positive mental health. The instrument comprises 14 positively worded items rated on a 5-point Likert scale (1 = “none of the time” to 5 = “all of the time”), producing total scores ranging from 14 to 70, with higher scores reflecting greater well-being. The Turkish adaptation of the WEMWBS was validated by Keldal (2015) in a sample of 371 adults aged 16 to 70. Consistent with the original version, factor-analytic findings supported a unidimensional structure, accounting for 51% of the variance. The scale demonstrated excellent internal consistency (Cronbach’s *α* = 0.92) and strong test–retest reliability over a one-week interval (*r* = 0.83). Criterion validity was evidenced through significant correlations with measures of subjective well-being, life satisfaction, and happiness, supporting its use as a reliable and valid indicator of psychological well-being in Turkish populations.

#### Connor-Davidson Resilience Scale (CD-RISC)

Resilience was assessed using the Connor–Davidson Resilience Scale (CD-RISC), a 25-item self-report instrument developed by Connor and Davidson (2003) to evaluate individuals’ capacity to cope with and adapt to adversity. Items are rated on a 5-point scale ranging from 0 (“not true at all”) to 4 (“true nearly all the time”), yielding total scores between 0 and 100. The original validation supported a five-factor structure encompassing personal competence, trust in one’s instincts, positive acceptance of change, sense of control, and spiritual influences. The Turkish validation of the CD-RISC was conducted by Karaırmak (2010) in a sample of 246 earthquake survivors. Exploratory and confirmatory factor analyses supported a three-factor model—tenacity and personal competence, tolerance of negative affect, and tendency toward spirituality—explaining 52% of the total variance. The scale demonstrated excellent internal consistency (Cronbach’s *α* = 0.92), with subscale reliabilities ranging from 0.79 to 0.93. Strong correlations with measures of self-esteem, hope, optimism, and affect further supported its construct validity within Turkish populations.

### Power analysis and sample size determination

*A priori* power analyses were conducted using G*Power 3.1 to determine the minimum sample size required for the planned statistical procedures. All calculations were based on two-tailed tests with an alpha level of 0.05 and desired statistical power of 1–*β* = 0.90. For independent-samples t tests, detecting a small-to-moderate effect size (Cohen’s d = 0.25) requires a total sample of approximately 500 participants under balanced group conditions. For one-way ANOVA designs with typical sociodemographic groupings (approximately four groups), identifying a small effect (*f* = 0.15) necessitates roughly 550 participants. For bivariate correlations, a small association (r = 0.15) similarly requires an estimated sample of 575 individuals. Multiple linear regression models with up to ten predictors require approximately 550–600 participants to detect a small-to-moderate effect increment (f^2^ = 0.03). The final sample size of N = 642 therefore met or exceeded all *a priori* estimates, ensuring ≥ 0.90 statistical power across the primary analyses involving depression, anxiety, PTSD symptoms, mental well-being, and resilience.

### Statistical analysis

All statistical analyses were performed using IBM SPSS Statistics (version 30). Prior to analysis, the dataset was screened for accuracy, missing values, and adherence to distributional assumptions. Descriptive statistics were used to summarize sociodemographic characteristics and psychological outcome variables. Group differences were examined using independent-samples t tests or one-way ANOVAs, with non-parametric alternatives applied when normality or homogeneity assumptions were violated. Bonferroni adjustments were used for *post hoc* comparisons, and chi-square tests were employed for analyses involving categorical variables. Effect sizes for all group comparisons (Cohen’s d for t tests and partial η^2^ for ANOVAs), along with 95% confidence intervals, are reported in the comparative tables to aid interpretation and account for family-wise error across multiple tests.

Associations among continuous variables were evaluated using Pearson correlation coefficients. Hierarchical multiple regression analyses were conducted to identify variables associated with mental well-being and resilience, while logistic regression was used to estimate the odds of meeting the threshold for probable PTSD (PCL-5 ≥ 47). All statistical tests were two-tailed with an alpha level of 0.05, and standardized effect size estimates (Cohen’s d, partial η^2^, odds ratios, standardized *β* coefficients) were reported.

Multicollinearity diagnostics were conducted before running the regression models. Variance inflation factors for depression, anxiety, and PTSD symptoms were all below 2.5, and tolerance values exceeded 0.40, indicating no problematic multicollinearity. Sensitivity analyses in which distress variables were entered separately produced comparable patterns of significance, further supporting the stability and robustness of the regression results.

Missing data were minimal (<3% per variable) and were handled using listwise deletion, resulting in complete-case analyses for all multivariate models. Consequently, sample sizes vary slightly across models depending on the availability of complete data for included predictors. Both R^2^ and adjusted R^2^ values are reported to provide more accurate estimates of explained variance.

To enhance methodological transparency and align the manuscript with reporting standards for observational research, a STROBE (Strengthening the Reporting of Observational Studies in Epidemiology) checklist and a participant-flow diagram summarizing recruitment, exclusions, and final sample size have been included in the Supplementary Table S5.

## Results

### Sample characteristics

The final sample comprised 642 adults residing in 11 cities affected by the 2023 Kahramanmaraş earthquakes. Participants ranged in age from 19 to 58 years (*M* = 35.4, SD = 10.8). Of the total sample, 346 individuals (53.9%) were female. Educational attainment varied, with 160 participants (24.9%) reporting primary education or below, 219 (34.1%) having completed high school, and 263 (41.0%) holding a university degree. Regarding marital status, 342 participants (53.3%) were married, 235 (36.6%) were single, and 65 (10.1%) were divorced. At the time of data collection, 418 participants (65.1%) were employed and 224 (34.9%) were unemployed. In terms of earthquake-related exposure, 371 participants (57.8%) reported experiencing direct losses or displacement, whereas 271 (42.2%) reported no major losses associated with the disaster.

### Psychological measures

Participants reported elevated levels of psychological distress across all standardized instruments. The mean BDI-II score was 22.1 (SD = 10.5), indicating moderate depressive symptomatology. Anxiety levels were similarly elevated, with a mean BAI score of 19.4 (SD = 9.2), corresponding to the mild-to-moderate range. PTSD symptoms were also substantial; the mean PCL-5 score of 40.0 (SD = 15.3) approached the recommended cutoff for probable PTSD.

In contrast, indicators of positive psychological functioning were notably lower than normative expectations. The mean WEMWBS score of 35.6 (SD = 9.7) was substantially below established population norms, which typically fall within the 47–50 range in the original UK validation and approximately 48.1 in the Turkish validation. This reflects an 11–14-point decrement relative to expected levels of well-being. Similarly, resilience scores were reduced: the mean CD-RISC score of 56.0 (SD = 14.2) fell below normative international values (70–82) as well as below Turkish norms for earthquake-exposed adults (62–65). Thus, the sample demonstrated diminished resilience alongside elevated distress nearly 2 years after the disaster.

All instruments exhibited excellent internal consistency in the current sample (Cronbach’s *α* = 0.90–0.94), aligning with reliability estimates reported in prior Turkish validation studies. Descriptive statistics and internal consistency coefficients for each measure are presented in Table 1.

**Table 1:** Descriptive statistics and reliability of psychological measures (*N* = 642).

Measure	Possible range	M (SD)	Cronbach’s *α*
Beck Depression Inventory–II (BDI-II)	0–63	22.1 (10.5)	0.90
Beck Anxiety Inventory (BAI)	0–63	19.4 (9.2)	0.93
PTSD Checklist – DSM-5 (PCL-5)	0–80	40.0 (15.3)	0.94
Warwick-Edinburgh Mental Well-Being Scale (WEMWBS)	14–70	35.6 (9.7)	0.92
Connor-Davidson Resilience Scale (CD-RISC)	0–100	56.0 (14.2)	0.92

### Group differences in psychological outcomes

Group comparisons across gender, marital status, education level, employment status, and trauma exposure are summarized in Table 2. Women reported significantly higher depressive symptoms than men (*p* = 0.032, d = 0.40), whereas no gender-based differences emerged for anxiety, PTSD symptoms, well-being, or resilience. Marital status was associated with anxiety levels, with single participants exhibiting higher anxiety scores than married individuals (*p* = 0.033); no additional marital-status differences were observed across the other outcomes.

**Table 2:** Group comparisons of psychological outcomes by gender, marital status, and trauma exposure.

Outcome	Gender (M vs. F)	Marital (Single vs. Married)	Trauma (No vs. Yes)
BDI-II	20.5 (11.0) vs. 23.7 (9.8), *p* = 0.032*	23.6 (9.9) vs. 21.1 (10.9), *p* = 0.144	19.1 (10.5) vs. 24.1 (10.1), *p* = 0.001**
BAI	18.4 (9.9) vs. 20.5 (8.3), *p* = 0.117	21.6 (8.9) vs. 18.4 (9.4), *p* = 0.033*	17.9 (9.6) vs. 20.5 (8.8), *p* = 0.046*
PCL-5	39.0 (15.5) vs. 41.0 (15.1), *p* = 0.376	43.4 (16.1) vs. 39.5 (14.9), *p* = 0.123	36.5 (15.4) vs. 42.3 (14.8), *p* = 0.009**
WEMWBS	36.4 (9.3) vs. 34.8 (10.0), *p* = 0.240	34.0 (10.7) vs. 36.2 (9.3), *p* = 0.164	37.4 (10.2) vs. 34.3 (9.1), *p* = 0.025*
CD-RISC	55.0 (14.4) vs. 57.0 (14.0), *p* = 0.318	55.0 (15.1) vs. 55.6 (14.0), *p* = 0.791	59.7 (15.0) vs. 53.5 (13.2), *p* = 0.002**

*, **indicates statistical significance at the *p* < 0.05 and *p* < 0.01 level (two-tailed).

Educational attainment showed a modest association with psychological functioning. Participants with university degrees demonstrated slightly higher resilience scores compared to those with primary education (*p* = 0.047). Trends toward significance were noted for depression and anxiety (both *p* ≈ 0.05); however, these associations did not remain significant in subsequent multivariate analyses. Employment status was unrelated to any of the outcomes assessed (all *p* > 0.50).

Trauma exposure was the most consistent and robust factor differentiating psychological outcomes. Participants who reported direct losses or displacement exhibited significantly higher depression (*p* = 0.001), anxiety (*p* = 0.046), and PTSD symptoms (*p* = 0.009), as well as lower well-being (*p* = 0.025) and resilience (*p* = 0.002), compared with those who did not experience major losses. These findings underscore the substantial psychological impact associated with severe earthquake-related exposure.

### Bivariate correlations

Pearson correlations among the psychological measures are shown in Table 3. Depression, anxiety, and PTSD symptoms were strongly interrelated (*r*s = 0.65–0.67, *p* < 0.001). Higher depression and PTSD scores were significantly associated with lower well-being (*r* = −0.54 and −0.63, respectively, *p* < 0.001). Resilience was inversely related to all distress measures (*r*s = −0.40 to −0.48, *p* < 0.001) and positively associated with well-being (*r* = 0.48, *p* < 0.001). All correlations were statistically significant, underscoring the consistent link between psychological distress and diminished well-being and resilience.

**Table 3:** Pearson correlation matrix for key psychological measures.

Variable	BDI-II	BAI	PCL-5	WEMWBS	CD-RISC
BDI-II	–	0.67**	0.65**	−0.54**	−0.46**
BAI	0.67**	–	0.65**	−0.48**	−0.40**
PCL-5	0.65**	0.65**	–	−0.63**	−0.48**
WEMWBS	−0.54**	−0.48**	−0.63**	–	0.48**
CD-RISC	−0.46**	−0.40**	−0.48**	0.48**	–

**indicates that the correlation is statistically significant at the *p* < 0.001 level (two-tailed).

**Figure 1:**
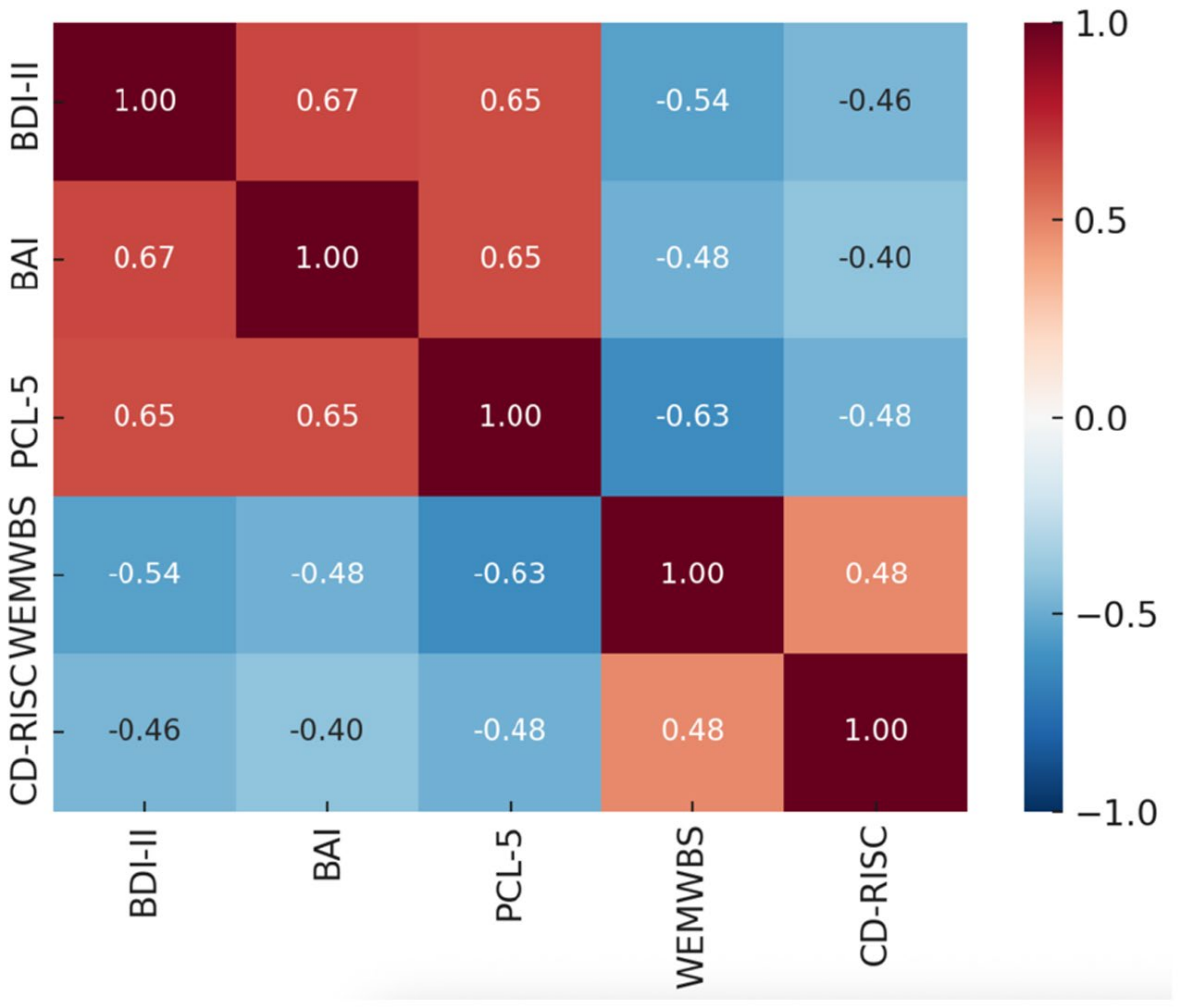
Heatmap Pearson correlation matrix of psychological measures.

The heatmap in Figure 1 displays Pearson correlations among depression (BDI-II), anxiety (BAI), PTSD symptoms (PCL-5), mental well-being (WEMWBS), and resilience (CD-RISC). Warmer colors indicate stronger positive associations, and cooler colors indicate stronger negative associations. All correlations were statistically significant (*p* < 0.001). Analyses and visualizations were generated using IBM SPSS Statistics 30.

**Figure 2:**
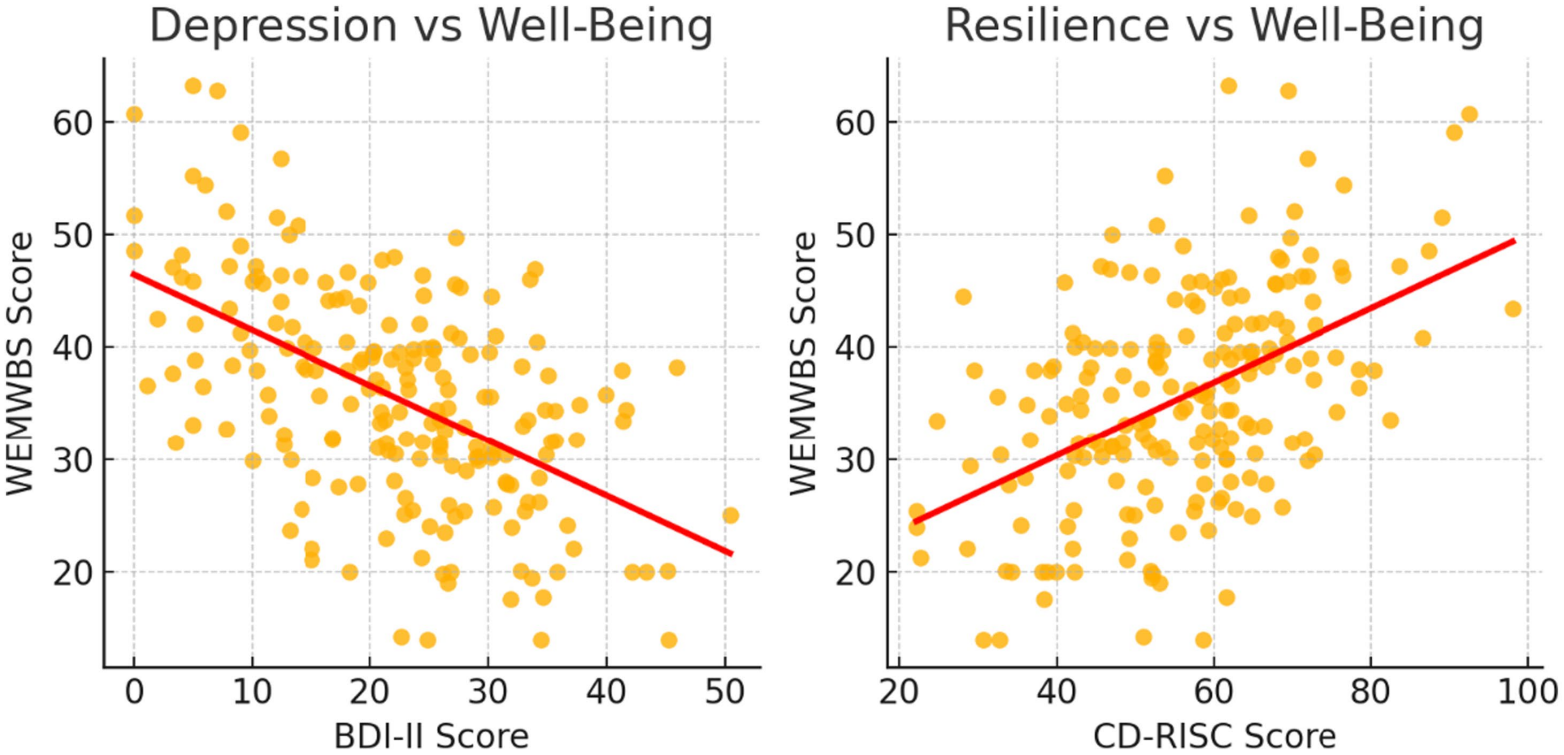
Scatterplots showing Pearson associations between distress and well-being.

Scatterplots in Figure 2 depict Pearson correlations between psychological distress variables (e.g., depression, anxiety, PTSD symptoms) and mental well-being (WEMWBS), with fitted regression lines for visualization. Analyses and visualizations were generated using IBM SPSS Statistics 30.

### Hierarchical regression predicting mental well-being

Results of the hierarchical regression are presented in Table 4. In Step 1, demographic and exposure variables explained 9% of the variance in well-being (*R^2^* = 0.09, *p* = 0.07), with no significant individual predictors. Trauma exposure showed a marginal effect (*β* = −0.11, *p* = 0.08), but gender, marital status, education, and employment were nonsignificant. Adding depression (BDI-II), anxiety (BAI), and PTSD symptoms (PCL-5) in Step 2 significantly improved the model (Δ*R^2^* = 0.36, *p* < 0.001). The final model accounted for 45% of the variance in well-being, *R^2^* = 0.45, *F* (9, 190) = 18.83, *p* < 0.001. Depression (*β* = −0.24, *p* = 0.005) and PTSD symptoms (*β* = −0.28, *p* = 0.001) independently predicted lower well-being, whereas anxiety was not significant (*β* = −0.09, *p* = 0.18). Demographic and exposure variables were no longer significant after distress measures were entered. These findings indicate that depressive and PTSD symptoms, rather than sociodemographic characteristics, were the primary determinants of diminished well-being. Full regression coefficients are presented in Supplementary Table S1.

**Table 4:** Hierarchical regression predicting mental well-being (WEMWBS).

Predictor	Step 1 *β*	*p*	Step 2 *β*	*p*
Age	−0.05	0.47	−0.02	0.78
Female (gender)	−0.06	0.30	−0.04	0.50
Married (vs. unmarried)	+0.04	0.53	+0.02	0.71
Education (ordinal)	+0.10	0.13	+0.07	0.24
Employed (vs. unemployed)	+0.03	0.73	+0.01	0.90
Trauma exposure (yes)	−0.11	0.08	−0.05	0.41
BDI-II (depression)	–	–	−0.24**	0.005
BAI (anxiety)	–	–	−0.09	0.18
PCL-5 (PTSD symptoms)	–	–	−0.28**	0.001

Model R^2^: 0.09 (Step 1), 0.45 (Step 2); Adjusted R^2^: 0.06 (Step 1), 0.43 (Step 2); ΔR^2^: 0.36*** (*p* < 0.001), added in Step 2; Model *N* = 642.

Standardized coefficients (*β*) reported. Adjusted R^2^ values added per reviewer recommendation. ΔR^2^ reflects variance explained by distress variables added in Step 2.

**Figure 3:**
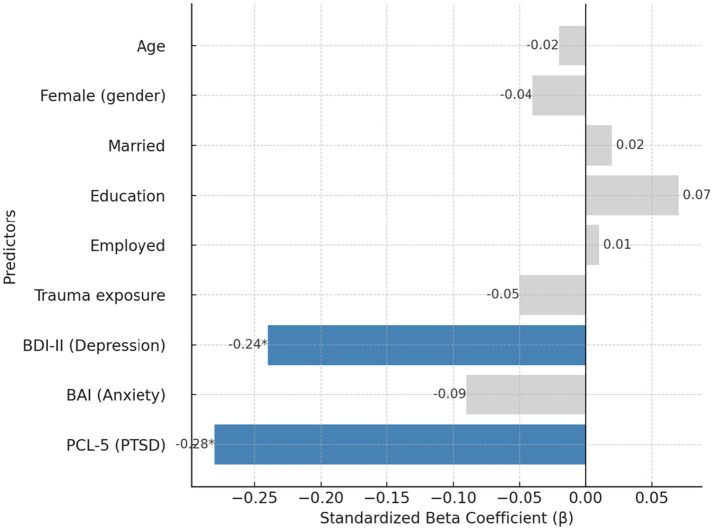
Predictors of mental well-being in hierarchical regression.

Figure 3 illustrates the standardized regression coefficients predicting mental well-being (WEMWBS). In the final model (*R*^2^ = 0.45), depression (*β* = −0.24, *p* = 0.005) and PTSD symptoms (*β* = −0.28, *p* = 0.001) were the only significant predictors of lower well-being. Anxiety and all demographic/exposure variables were nonsignificant, indicating that clinical symptom burden, rather than background characteristics, primarily determined survivors’ well-being. Analyses and visualizations were conducted using IBM SPSS Statistics 30.

### Hierarchical regression predicting resilience

Results are shown in Table 5. In Step 1, demographic and exposure variables accounted for only 7% of the variance in resilience (*R*^2^ = 0.07, *p* = 0.12), with no significant predictors. Weak trends for education (*β* = 0.12, *p* = 0.06) and trauma exposure (*β* = −0.10, *p* = 0.11) did not reach significance. In Step 2, adding depression, anxiety, and PTSD symptoms significantly improved the model (Δ*R*^2^ = 0.25, *p* < 0.001). The final model explained 32% of the variance (*R*^2^ = 0.32, *F* (9,190) = 10.76, *p* < 0.001). Depression (*β* = −0.22, *p* = 0.004) and PTSD symptoms (*β* = −0.26, *p* = 0.001) were significant negative predictors, while anxiety was nonsignificant (*β* = −0.07, *p* = 0.25). All demographic and exposure variables, including education, were nonsignificant once psychological distress was considered. These results demonstrate that resilience is primarily determined by current levels of depression and PTSD, not by background sociodemographic characteristics. Detailed coefficients are reported in Supplementary Table S2.

**Table 5:** Hierarchical regression predicting resilience (CD-RISC).

Predictor	Step 1 *β*	*p*	Step 2 *β*	*p*
Age	+0.02	0.78	+0.04	0.62
Female (gender)	+0.06	0.33	+0.08	0.22
Married (vs. unmarried)	+0.04	0.52	+0.03	0.65
Education (ordinal)	+0.12	0.06	+0.06	0.30
Employed (vs. unemployed)	+0.01	0.90	+0.00	0.98
Trauma exposure (yes)	−0.10	0.11	−0.04	0.54
BDI-II (Depression)	–	–	−0.22**	0.004
BAI (Anxiety)	–	–	−0.07	0.25
PCL-5 (PTSD symptoms)	–	–	−0.26**	0.001

Model R^2^: 0.07 (Step 1), 0.32 (Step 2); Adjusted R^2^: 0.04 (Step 1), 0.30 (Step 2); ΔR^2^: 0.25*** (*p* < 0.001), added in Step 2; Model *N* = 642.

CD-RISC = Connor–Davidson Resilience Scale. Adjusted R^2^ values added per reviewer recommendation.

**Figure 4:**
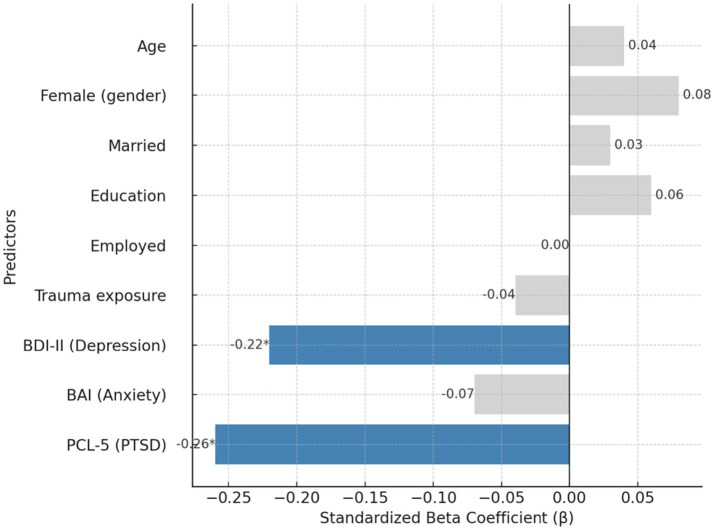
Predictors of resilience (CD-RISC) in hierarchical regression.

Figure 4 displays the standardized regression coefficients predicting psychological resilience. In the final model (*R*^2^ = 0.32), depression (*β* = −0.22, *p* = 0.004) and PTSD symptoms (*β* = −0.26, *p* = 0.001) were significant negative predictors of resilience. Anxiety and all demographic/exposure variables were nonsignificant, indicating that current clinical symptoms—not background characteristics—were the primary determinants of resilience. Analyses and visualizations were generated using IBM SPSS Statistics 30.

### Logistic regression predicting probable PTSD diagnosis

A logistic regression was conducted to identify predictors of probable PTSD, defined as a PCL-5 score ≥ 47. The overall model was statistically significant, χ^2^ (8) = 58.9, *p* < 0.001, with good fit (Nagelkerke *R^2^* = 0.50) and an overall classification accuracy of 82% (sensitivity = 80%, specificity = 83%). Results are shown in Table 6. Depressive symptoms were the strongest predictor: each one-point increase on the BDI-II was associated with a 10% increase in the odds of meeting PTSD criteria (OR = 1.10, 95% CI [1.04–1.16], *p* = 0.001). Female gender was also a significant predictor (OR = 1.80, 95% CI [1.08–2.99], *p* = 0.024), consistent with prior findings of elevated PTSD risk among women. No other demographic variables (age, marital status, education, employment) or trauma exposure independently predicted PTSD when controlling for psychological symptoms. Anxiety symptoms were also nonsignificant (OR = 1.02, 95% CI [0.98–1.06], *p* = 0.298). The complete regression model is provided in Supplementary Table S3.

**Table 6:** Logistic regression predicting probable PTSD diagnosis (PCL-5 ≥ 47).

Predictor	Odds ratio (OR)	95% CI for OR	*p*
Female (vs. male)	1.80	1.08–2.99	0.024*
Age (years)	0.99	0.96–1.02	0.501
Married (vs. unmarried)	0.78	0.50–1.23	0.279
Education level (ordinal)	0.90	0.75–1.08	0.338
Employed (vs. unemployed)	1.02	0.60–1.72	0.947
Trauma exposure (yes)	1.23	0.83–1.83	0.307
BDI-II (Depression)	1.10	1.04–1.16	0.001**
BAI (Anxiety)	1.02	0.98–1.06	0.298

Model *χ*^2^ (8) = 58.9, *p* < 0.001; Nagelkerke R^2^ = 0.50; classification accuracy = 82%. OR > 1 indicates higher odds of PTSD diagnosis.

*, **indicates statistical significance at the *p* < 0.05 and *p* < 0.01 level (two-tailed).

These findings indicate that depressive symptom severity and female gender significantly increased the likelihood of probable PTSD, while other background factors did not add explanatory power beyond current psychological distress. Beyond accuracy, sensitivity, and specificity, the logistic model demonstrated strong discriminative performance, with an area under the ROC curve of 0.87 (95% CI: 0.84–0.90). Calibration was adequate, as indicated by a non-significant Hosmer–Lemeshow goodness-of-fit test (*p* > 0.05), and graphical inspection of predicted versus observed probabilities showed no systematic miscalibration.

**Figure 5:**
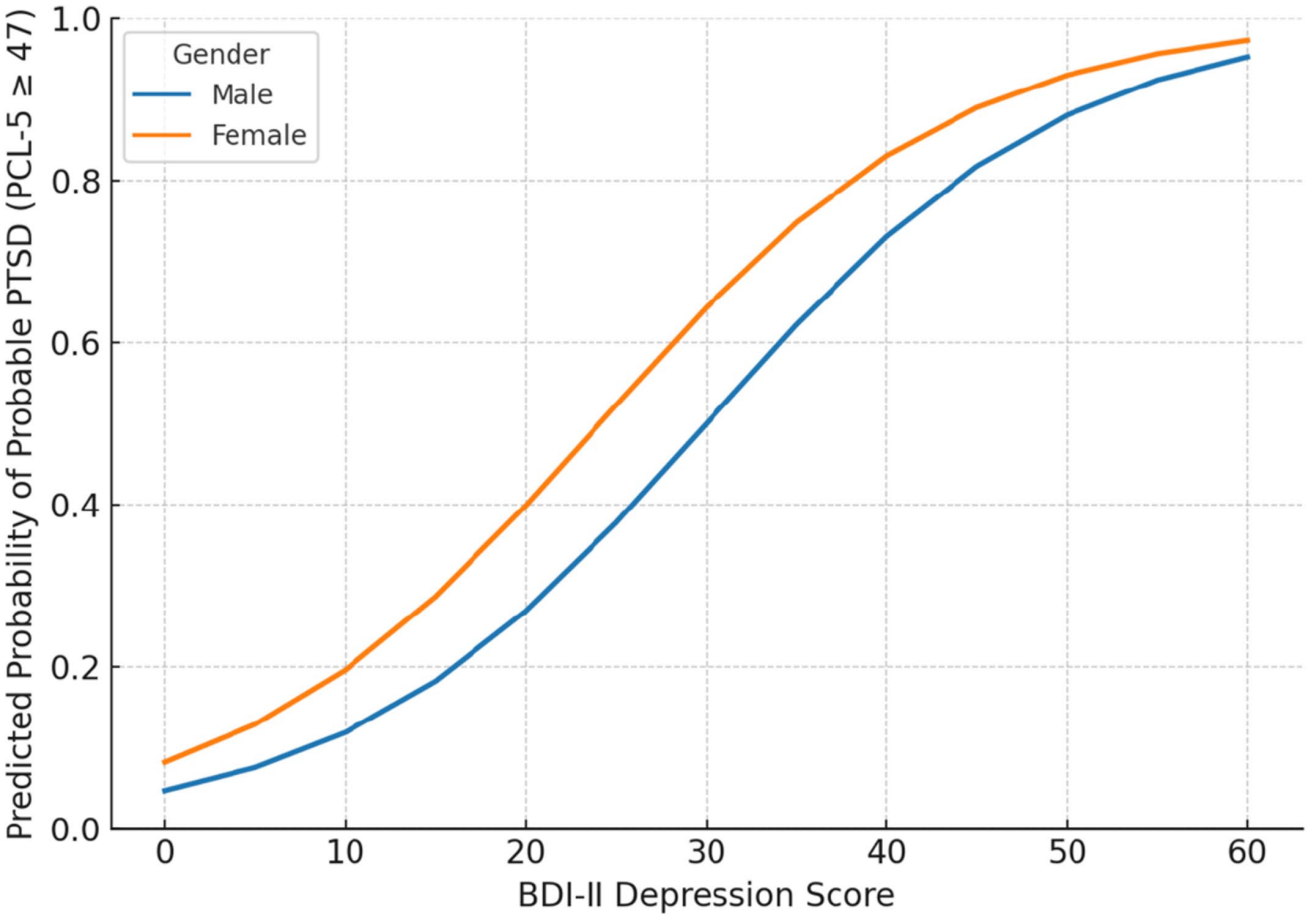
Predicted probability of probable PTSD (PCL-5 ≥ 47) by gender and depression severity.

Figure 5 shows the predicted probabilities of meeting the PTSD cut-off (PCL-5 ≥ 47) across levels of depression severity (BDI-II), stratified by gender. Higher depression scores markedly increased PTSD risk, and female survivors consistently exhibited higher predicted probabilities than males at comparable symptom levels. These findings correspond to the logistic regression results, where depression (OR = 1.10, *p* = 0.001) and female gender (OR = 1.80, *p* = 0.024) were significant predictors, while trauma exposure and other demographic variables were nonsignificant. Analyses and visualizations were generated using IBM SPSS Statistics 30.

Overall, 31% of participants scored above the PCL-5 cut-off for probable PTSD, and mean scores indicated moderate levels of depression and anxiety. Consistent with the Turkish validation of the PCL-5 (Boysan et al., 2017), the ≥47 threshold was used to define probable PTSD. Using this cut-off, 31.0% of participants screened positive for probable PTSD (95% CI: 27.4–34.8%). To assess the robustness of prevalence estimates, a sensitivity analysis was conducted using a more inclusive threshold (PCL-5 ≥ 33). Under this criterion, 48.9% of participants screened positive (95% CI: 45.0–52.7%). Logistic regression results remained substantively unchanged when re-estimated with the lower threshold, indicating that model inferences were stable across cut-off values. Female participants and those exposed to direct trauma had significantly higher symptom levels. Well-being and resilience were inversely associated with PTSD and depression, and multivariate models showed that these distress variables explained substantially more variance than demographic characteristics.

## Discussion

In this multi-site cross-sectional study of adults who survived the 2023 Kahramanmaraş earthquakes, the findings provide compelling evidence of both the substantial psychological burden imposed by the disaster and the important associations between resilience, well-being, and post-disaster adjustment. Consistent with expectations and with prior research on major earthquakes, levels of psychological distress were markedly elevated across the sample. A considerable proportion of participants reported clinically significant symptoms of PTSD, depression, and anxiety, and the results suggest that many survivors continued to experience pronounced distress nearly 2 years after the event. This pattern aligns with early reports from the affected region indicating that more than half of adults exposed to the February 2023 earthquakes may experience PTSD-related symptoms (Lok et al., 2025; Yılmaz and Doğan, 2025). Elevated levels of depression and anxiety accompanied the PTSD burden, reflecting the broad and persistent spectrum of trauma-related psychopathology frequently observed following large-scale catastrophic events.

Despite the growing body of research conducted in the immediate aftermath of the February 2023 Kahramanmaraş earthquakes, there remains a notable lack of large-scale, theoretically grounded studies examining survivors’ long-term psychological adjustment. The present study makes several unique contributions to this emerging literature. First, it represents one of the largest multi-site assessments of adult survivors nearly 2 years after the disaster, capturing persistent patterns of distress, resilience, and well-being across 11 heavily affected cities. This design provides a rare and timely perspective on the prolonged psychological impact of the earthquake sequence, extending beyond the short-term reactions documented in earlier reports. Second, by simultaneously evaluating psychological distress, resilience, and well-being within a unified theoretical framework, the study moves beyond traditional symptom-focused disaster research and offers a more holistic understanding of mental health during protracted recovery. Third, the use of rigorous multivariate models enables the identification of the factors most strongly associated with long-term adjustment, highlighting the central role of depression and PTSD symptoms compared with demographic or exposure variables. Together, these contributions advance the field by providing high-quality, late-phase evidence on the psychological consequences of the 2023 earthquakes and by underscoring the importance of integrating both negative and positive mental health constructs in post-disaster research.

Importantly, beyond these adverse outcomes, the findings also revealed substantial impairments in positive mental health: overall well-being scores were considerably lower than normative benchmarks, and many survivors reported difficulties experiencing positive affect or life satisfaction in the aftermath of the disaster (Kaplan et al., 2024). Taken together, these results highlight that the psychological consequences of the earthquakes extend well beyond diagnosable psychiatric disorders, affecting survivors’ broader psychosocial functioning and perceived quality of life. Notably, even individuals who did not meet criteria for probable PTSD or clinically significant depression exhibited reduced well-being, underscoring the need for comprehensive, multidimensional approaches to post-disaster recovery.

The examination of group differences provided insight into which subgroups of survivors were most psychologically affected. Consistent with prior disaster research, female participants reported significantly higher depressive symptoms than males, although gender differences were not observed for PTSD or anxiety in the unadjusted comparisons (Fu et al., 2021; Cénat and Derivois, 2014). Notably, when multivariate models accounted for overall symptom burden, female gender emerged as a significant predictor of probable PTSD, indicating an elevated risk for meeting diagnostic thresholds among women. This pattern has been widely documented in post-disaster contexts and may reflect intersecting biological, social, and caregiving-related factors that heighten vulnerability among women (Khatri et al., 2019).

Trauma exposure severity was also strongly associated with psychological outcomes. Survivors who experienced life-threatening or highly distressing events—such as entrapment under collapsed structures, severe injury, the loss of immediate family members, or witnessing extensive destruction—reported substantially higher PTSD, depression, and anxiety symptoms than those with less severe exposure (Wu et al., 2014). These results are consistent with the dose–response pattern frequently described in disaster literature, whereby greater exposure to traumatic stressors is associated with greater psychological distress. In the present sample, individuals who lost first-degree relatives or whose homes were entirely destroyed were among those with the highest levels of symptom severity, a finding that aligns with emerging evidence from the 2023 earthquakes.

By contrast, survivors who did not experience major personal losses—such as bereavement or significant property destruction—reported relatively lower, though still elevated, levels of psychological symptoms. Importantly, the analyses revealed limited evidence that demographic variables such as age, education, marital status, or employment played a major role in differentiating psychological outcomes, a pattern also noted in other studies of large-scale disasters where symptoms remain high across groups regardless of socioeconomic status. Although age has been identified as a risk factor in some contexts—with both older and younger adults reported as more vulnerable in different studies—the present study did not find age to be a significant predictor of distress, potentially reflecting the complex interaction of risk and resilience factors within this cohort (Kun et al., 2013; Kongshøj and Berntsen, 2023). Taken together, these subgroup findings indicate that women—particularly in adjusted models—and individuals with severe earthquake-related exposure bear a disproportionate psychological burden. These observations underscore the need to prioritize these groups in post-disaster mental health interventions.

Beyond characterizing overall distress levels, a central aim of this study was to identify factors associated with key psychological outcomes—namely PTSD severity, mental well-being, and resilience. The regression analyses yielded several important observations. Consistent with expectations, trauma-related variables demonstrated strong associations with PTSD symptom severity in the bivariate analyses. Survivors who experienced highly distressing events, such as bereavement or entrapment under rubble, exhibited significantly higher PCL-5 scores, in line with previous evidence indicating that such events constitute potent risk factors for post-traumatic stress (Chan et al., 2012). However, in the multivariate logistic regression model, trauma exposure did not remain a statistically significant independent predictor after accounting for psychological symptom variables. Instead, female gender and depressive symptom severity emerged as the most robust predictors of probable PTSD, underscoring their salience as markers of elevated vulnerability.

Although resilience was not explicitly examined as a predictor of probable PTSD in the multivariate models, the bivariate analyses revealed a strong inverse association between resilience and PTSD symptoms. This finding aligns with theoretical frameworks (Thompson et al., 2018) and longitudinal research demonstrating that higher resilience—characterized by optimism, adaptive coping strategies, and strong social support—is associated with reduced PTSD symptom severity (Kamphuis et al., 2021; Prati and Pietrantoni, 2009; Weinberg et al., 2016). While causal inferences cannot be drawn from the cross-sectional design, the observed pattern is consistent with evidence suggesting that resilience may play a protective role in mitigating the long-term psychological effects of traumatic events.

When predictors of mental well-being, as measured by WEMWBS scores, were examined, the analyses revealed a pattern that closely aligned with the findings for PTSD. Survivors with lower levels of psychological distress—specifically fewer symptoms of PTSD, depression, and anxiety—reported significantly higher well-being. PTSD symptom severity demonstrated a strong inverse association with well-being: individuals with minimal PTSD symptoms exhibited the highest levels of psychological well-being, whereas those with severe symptoms scored markedly lower on the WEMWBS. Consistent with this relationship, the regression analyses indicated that lower PTSD and depressive symptom levels were significant predictors of higher well-being, underscoring the close link between distress reduction and improvements in subjective mental health.

However, distress reduction was not the sole determinant of well-being. Resilience again emerged as an independent positive correlate. Even after accounting for psychological symptoms, individuals with higher resilience reported substantially greater well-being. This suggests that resilience supports more than the absence of psychopathology; it also contributes meaningfully to the presence of positive mental health attributes such as optimism, purpose, and life satisfaction. One possible explanation is that resilient survivors may draw upon adaptive coping strategies and psychological resources that allow them to maintain or restore well-being despite ongoing adversity. A modest trend was also observed in which older age was associated with slightly higher well-being (and lower PTSD), although this association did not reach strong statistical significance in the fully adjusted models. If substantiated in future research, this trend may reflect the influence of accumulated life experience or previous exposure to hardship, as some studies have suggested (Yayla et al., 2025; Ardalan et al., 2011). Nevertheless, given the mixed evidence regarding age-related differences in disaster contexts, additional research is warranted. Overall, these findings highlight the critical role of psychosocial resources—particularly resilience, and the social support elements embedded within it—in promoting positive adaptation. At the same time, they reinforce that elevated distress is intrinsically linked to diminished well-being, emphasizing the need for interventions that simultaneously reduce symptoms and strengthen survivors’ resilience capacities.

Predictors of resilience were also examined. Although the cross-sectional design precludes causal inference, the correlational analyses revealed a consistent pattern: resilience was negatively associated with all indicators of psychological distress and positively associated with mental well-being. Survivors who reported higher resilience tended to exhibit lower levels of PTSD, depression (BDI-II), and anxiety (BAI), alongside higher WEMWBS scores. These findings suggest that resilience, distress, and well-being lie on interconnected continua, such that individuals functioning well in one domain often demonstrate strengths in the others. It is plausible that resilience both shapes and is shaped by mental health. For example, individuals with higher resilience may possess coping skills, adaptive cognitive appraisals, or stronger support networks that enable them to navigate post-disaster challenges more effectively, thereby reducing psychological symptoms. Conversely, lower levels of distress may allow survivors to better mobilize and sustain their resilience resources. Demographic factors showed limited explanatory value in this context; aside from a non-significant trend toward slightly higher resilience among males, variables such as age, education, marital status, and employment did not meaningfully differentiate resilience levels. Instead, unmeasured experiential and psychosocial variables—such as perceived social support, coping behaviors, and processes of meaning-making—are likely to play a more prominent role in shaping resilience (Theron and Theron, 2014; Çınaroğlu, 2024). Overall, the central implication is that resilience is closely intertwined with psychological outcomes. Strengthening survivors’ resilience capacities may therefore represent a key pathway for enhancing both symptom recovery and broader well-being in the aftermath of large-scale disasters.

The present findings align closely with the broader literature on disaster mental health and carry several important implications for both intervention and theoretical models. First, the high prevalence of PTSD and depressive symptoms observed in this sample confirms that mental health services must constitute a central component of disaster response efforts following the 2023 earthquakes. The psychological burden in the affected region remains substantial, with evidence pointing to widespread trauma- and grief-related reactions nearly 2 years after the event. This underscores the need for comprehensive, large-scale mental health interventions—including systematic screening and outreach initiatives to identify individuals experiencing significant PTSD, anxiety, or depression, followed by accessible and appropriate treatment options. Trauma-focused therapies, such as Trauma-Focused Cognitive Behavioral Therapy and Eye Movement Desensitization and Reprocessing, should be prioritized for survivors with severe PTSD, whereas psychoeducation and supportive counseling may be effective for individuals with moderate levels of distress (Leiva-Bianchi et al., 2018; Saltini et al., 2018). The findings also suggest that certain subgroups warrant particular attention. Women, bereaved individuals, and those who sustained severe injuries or displacement appear to be at heightened risk and may benefit from proactive monitoring and early therapeutic intervention. Culturally sensitive approaches that incorporate locally validated tools and take into account community norms surrounding emotional expression are essential for promoting engagement and enhancing treatment acceptability (Çınaroğlu, 2025). Given the scale of mental health needs and the limitations in available professional capacity, scalable and resource-efficient strategies are critical. Psychological First Aid (PFA), delivered by trained non-specialists, has demonstrated value in similar disaster contexts, and was deployed widely in Türkiye after the earthquakes. The present findings highlight the need to sustain and expand such support. Over the medium and long term, community-based programs—including peer support groups, community health worker outreach, and integration of mental health services into primary care—may contribute to ongoing recovery.

Evidence from prior large-scale disasters supports the importance of community resources and social connectedness. Experiences from previous Turkish earthquakes, including the 1999 Marmara quake, show that strengthening social networks and mobilizing community structures can substantially mitigate trauma-related outcomes. Enhancing social connectedness through community centers, group activities, or commemorative gatherings may help counteract isolation and hopelessness, both of which contribute to chronic PTSD and depression. Observations from other major crises indicate that disaster-related mental health needs often persist long after the initial emergency phase and may result in sustained increases in service utilization; for example, Barlattani et al. (2024) reported prolonged elevations in psychiatric admissions following both the L’Aquila earthquake and the COVID-19 lockdown. Situating the current findings within this larger context suggests that the considerable symptom burden and reduced resilience observed nearly 2 years after the 2023 Kahramanmaraş earthquakes are likely to generate extended mental health service needs. These insights reinforce the importance of implementing integrated long-term trauma care, proactive outreach, and resilience-building interventions to ensure continuity of support throughout the prolonged course of post-disaster recovery.

Beyond the treatment of psychopathology, the present findings underscore the importance of interventions aimed at strengthening resilience and enhancing well-being as integral components of holistic post-disaster recovery. The observed association between resilience and more favorable psychological outcomes indicates that fostering resilience is not merely an abstract or long-term ideal but a practical and attainable intervention target with meaningful implications for survivors’ mental health. Strategies designed to enhance resilience may include structured training programs focused on problem-solving, emotion regulation, and stress-management skills, which can be implemented through brief workshops or group-based formats within temporary housing communities.

Strengthening familial and social support systems represents another important avenue for intervention. Approaches that incorporate family members or peer-based models—such as buddy systems, supportive listening groups, or peer mentoring—may reinforce survivors’ sense of connectedness and belonging, thereby bolstering their resilience. Evidence from prior research suggests that even low-intensity interventions, such as facilitating communication with social networks through mobile devices or organizing community gatherings, can produce measurable improvements in well-being following disasters (Bryant et al., 2017). Psychoeducation focused on common post-traumatic reactions and the normalization of help-seeking can further empower survivors and reduce feelings of helplessness (Hisli Sahin et al., 2011).

From a public health standpoint, collaboration with community leaders and leveraging local cultural strengths—such as spiritual coping practices, communal rituals, and traditions of mutual aid—may amplify resilience at the community level. Consistent with findings from this study, survivors have reported relying on prayer and social support as primary coping strategies, a pattern also observed by Akçay et al. (2024). Integrating such culturally meaningful coping practices into mental health programming—for example, by providing spaces for collective prayer, remembrance activities, or community-based support meetings alongside psychological services—may enhance engagement, cultural acceptability, and overall intervention effectiveness.

In terms of theoretical implications, the dual emphasis on psychological distress and positive mental health aligns closely with contemporary conceptualizations of mental health. The findings lend support to the “dual continuum” framework, which posits that mental health encompasses both the absence of mental illness and the presence of positive well-being. This distinction was evident in the current sample; some survivors who exhibited relatively low levels of clinical symptoms nonetheless reported diminished well-being, whereas others with high levels of well-being endorsed fewer symptoms. Such patterns illustrate that these dimensions, while related, are distinct constructs that must be assessed and addressed in parallel to obtain a comprehensive understanding of post-disaster functioning. The inclusion of both WEMWBS and CD-RISC in this study therefore provides a more nuanced and multidimensional picture of survivor outcomes than would reliance on PTSD or depression measures alone.

The results also highlight the interaction between risk factors (e.g., trauma severity, female gender) and protective factors (e.g., resilience, social support) in shaping psychological trajectories following disaster exposure. This dynamic interplay is consistent with ecological models of disaster mental health and aligns with Norris et al.’s disaster response framework (Norris et al., 2008), which emphasizes that both resource loss and resource gain processes contribute to psychological outcomes. Although resilience was not included as a direct predictor of PTSD in the multivariate models, its strong correlations with both distress and well-being reinforce theoretical models of resilience and post-traumatic growth, suggesting that interventions fostering meaning-making, coping self-efficacy, and empowerment may promote not only symptom reduction but also positive psychological development after trauma.

Additionally, the findings add to ongoing discussions regarding the role of sociocultural factors in disaster recovery. Although the present analyses did not identify strong effects for variables such as education, marital status, or employment, other factors—such as ethnicity or income—were not assessed. Prior work (e.g., Kizilhan et al., 2024) suggests that in large-scale catastrophes, psychosocial impacts may cut across conventional socioeconomic boundaries, with elevated symptoms observed broadly across population groups. This phenomenon may reflect a “shared trauma” effect, in which communal exposure produces a more uniform distribution of psychological consequences. Further research is needed to examine these sociocultural processes and their implications for theory and intervention.

### Limitations

Despite its strengths, this study has several limitations. First, the cross-sectional design provides only a snapshot nearly 2 years after the earthquakes, precluding causal inferences and leaving the trajectory of psychological symptoms and resilience unknown. Longitudinal follow-up is needed to clarify directionality (e.g., whether resilience buffers PTSD or vice versa) and to detect delayed-onset disorders. Second, although the sample was large and drawn from 11 heavily affected cities, the sites differed in the degree of destruction, displacement, and access to resources, creating natural heterogeneity in survivor experiences. The analyses did not model clustering by city, and therefore city-level differences may have influenced psychological outcomes; this should be considered when interpreting the findings. Third, participation was voluntary and limited to individuals accessible in affected areas, which may exclude survivors who relocated or were severely impaired. Fourth, all data were based on self-report questionnaires. Although validated instruments were used, biases such as underreporting, overreporting, and shared method variance remain possible, and prevalence estimates reflect probable—rather than clinically diagnosed—conditions. Fifth, contextual factors such as social support, coping strategies, community resources, and pre-disaster mental health were not directly measured, limiting the ability to account for their effects. Another important limitation concerns the lack of a quantifiable exposure-severity index. Although the study distinguished between participants with and without major earthquake-related losses, exposure was captured in a binary format and did not include graded measures of humanitarian, financial, structural, or social loss (e.g., extent of property destruction, financial damage, duration of displacement, or loss of livelihood). This limits the ability to examine dose–response relationships or determine which specific types or intensities of earthquake-related adversity are most strongly associated with depression, anxiety, PTSD symptoms, resilience, and well-being. As a result, interpretations of exposure effects should be viewed with caution, and future research would benefit from employing validated multi-domain exposure-severity instruments. The decision to use a simplified, dichotomous exposure indicator was also influenced by the practical constraints of conducting multi-site fieldwork nearly 2 years after the earthquakes. Because data were collected across 11 cities with substantial variability in destruction, displacement, and recovery conditions, obtaining standardized, objective, and multi-domain exposure metrics (e.g., degree of property damage, financial loss, duration of homelessness, or detailed bereavement characteristics) was not feasible in a consistent manner across sites. To preserve comparability and measurement integrity across all data-collection locations, a binary exposure classification was adopted. This rationale is now explicitly stated to provide clarity regarding the methodological choices. Another limitation relates to the restricted sampling frame. Individuals with current psychiatric diagnoses, psychiatric medication use, or ongoing psychotherapy were excluded to focus on untreated, community-dwelling survivors. However, these criteria inadvertently removed high-risk groups who are more likely to exhibit severe PTSD, depression, anxiety, and reduced well-being. As a result, the estimates presented in this study likely underestimate the true psychological burden among the broader survivor population, and the findings should be interpreted with this reduced generalisability in mind. Also, although regression models identified statistical associations between distress variables, well-being, and resilience, the cross-sectional design precludes any causal inference. The temporal ordering of symptoms and positive psychological resources cannot be established, and all observed relationships should be interpreted as correlations rather than causal effects. Longitudinal designs are needed to determine directionality and examine potential causal pathways. Finally, the focus on adults restricts generalizability to children, adolescents, or other vulnerable groups such as the elderly or individuals with disabilities. Future research should incorporate validated, multi-domain exposure-severity instruments to enable more granular modeling of disaster-related adversity and to clarify how specific forms and intensities of loss influence psychological distress, resilience, and well-being.

## Conclusion

This study offers a comprehensive and nuanced portrayal of the psychological aftermath of the 2023 Kahramanmaraş earthquakes. The findings indicate that nearly 2 years after the disaster, a substantial proportion of survivors continue to experience elevated levels of trauma-related distress, highlighting an ongoing public mental health challenge that requires sustained attention. At the same time, the results also reveal meaningful signs of resilience; many individuals demonstrate the capacity to adapt, recover, or maintain aspects of well-being despite prolonged adversity.

These dual observations underscore the need for a balanced, two-pronged approach to post-disaster mental health care. On the one hand, accessible and evidence-based clinical services remain essential for survivors experiencing significant PTSD, depression, or anxiety. On the other hand, preventive and strengths-oriented interventions aimed at enhancing resilience and well-being are equally critical. Mental health support will be most effective when it addresses both symptom reduction and the promotion of adaptive psychological resources, including coping efficacy, social connectedness, and meaning-making.

As Türkiye progresses through a prolonged recovery process, the integration of mental health services into broader rebuilding efforts—ranging from primary care settings in temporary housing areas to community-led support initiatives—will be vital. The present findings support such integrated models and offer empirical guidance for targeted outreach, particularly for subgroups at elevated risk, such as women, bereaved individuals, and survivors with severe exposure.

Ultimately, promoting psychological recovery extends beyond mitigating distress; it involves restoring hope, agency, and social cohesion among survivors. By identifying key correlates of both vulnerability and resilience, this study contributes to the evidence base needed to inform long-term disaster mental health strategies. Continued investment in research, service capacity, and community-based interventions will be essential for fostering sustained recovery and supporting affected populations as they work toward renewed well-being.

## Data Availability

The original contributions presented in the study are included in the article/Supplementary material, further inquiries can be directed to the corresponding author.
