# Supplementary material for: Psychological distress, resilience, and well-being among survivors of the 2023 Kahramanmaraş earthquakes: a multi-site cross-sectional study
Source: Front Psychol. 2025 Dec 10;16:1730083. doi: 10.3389/fpsyg.2025.1730083 (PMC12727615; doi:10.3389/fpsyg.2025.1730083)
Supplement: Supplementary file 1 [file Data_Sheet_1.pdf]

Supplementary Table S1. Hierarchical regression predicting mental well-being (WEMWBS)

**Predictor**	**B**	**SE**	**β**	**95% CI (Lower–Upper)**	**p**
Age	–0.03	0.09	–.02	–0.20, 0.14	.78
Female (gender)	–0.45	0.67	–.04	–1.77, 0.87	.50
Married (vs. unmarried)	+0.23	0.63	+.02	–1.01, 1.47	.71
Education (ordinal)	+0.31	0.27	+.07	–0.22, 0.84	.24
Employed (vs. unemployed)	+0.12	0.92	+.01	–1.70, 1.94	.90
Trauma exposure (yes)	–0.87	1.05	–.05	–2.94, 1.20	.41
BDI-II (Depression)	–0.28	0.10	–.24	–0.48, –0.08	.005
BAI (Anxiety)	–0.12	0.09	–.09	–0.30, 0.06	.18
PCL-5 (PTSD)	–0.19	0.06	–.28	–0.31, –0.07	.001

Supplementary Table S2. Hierarchical regression predicting resilience (CD-RISC)

**Predictor**	**B**	**SE**	**β**	**95% CI (Lower–Upper)**	**p**
Age	+0.05	0.09	+.04	–0.12, 0.22	.62
Female (gender)	+0.59	0.76	+.08	–0.90, 2.08	.22
Married (vs. unmarried)	+0.33	0.72	+.03	–1.09, 1.75	.65
Education (ordinal)	+0.42	0.38	+.06	–0.33, 1.17	.30
Employed (vs. unemployed)	+0.08	0.97	+.00	–1.84, 2.00	.98

Trauma exposure (yes)	–0.54	0.89	–.04	–2.28, 1.20	.54
BDI-II (Depression)	–0.25	0.09	–.22	–0.43, –0.07	.004
BAI (Anxiety)	–0.08	0.07	–.07	–0.22, 0.06	.25
PCL-5 (PTSD)	–0.15	0.04	–.26	–0.23, –0.07	.001

Supplementary Table S3. Logistic regression predicting probable PTSD (PCL-5 ≥ 47)

**Predictor**	**B**	**SE**	**OR**	**95% CI for OR**	**p**
Female (vs. male)	0.59	0.26	1.80	1.08 – 2.99	.024
Age (years)	–0.01	0.02	0.99	0.96 – 1.02	.50
Married (vs. unmarried)	–0.25	0.23	0.78	0.50 – 1.23	.28
Education (ordinal)	–0.10	0.11	0.90	0.75 – 1.08	.34
Employed (vs. unemployed)	0.02	0.28	1.02	0.60 – 1.72	.95
Trauma exposure (yes)	0.21	0.21	1.23	0.83 – 1.83	.31
BDI-II (Depression)	0.10	0.03	1.10	1.04 – 1.16	.001
BAI (Anxiety)	0.02	0.02	1.02	0.98 – 1.06	.30

Supplementary Analysis Table S4: Exploratory Mediation of the Distress → Well-Being Association by Resilience

To address the reviewer's suggestion and to explore the positive-psychology implications of the findings, an exploratory mediation analysis was conducted to examine whether psychological resilience (CD-RISC) partially mediates the association between psychological distress and mental well-being (WEMWBS). Because mediation cannot be causally inferred from cross-sectional data, results are interpreted descriptively.

Two indicators of distress were tested separately as predictors: depression (BDI-II) and PTSD symptoms (PCL-5). Resilience (CD-RISC) served as the mediator, and mental well-being (WEMWBS) was the outcome. Bootstrapped indirect effects (5,000 samples, bias-corrected) were estimated.

Results:
Both models showed significant indirect effects through resilience:

- BDI-II → Resilience → Well-being: Indirect effect = –0.11, 95% CI [–0.18, –0.06]
- PCL-5 → Resilience → Well-being: Indirect effect = –0.10, 95% CI [–0.17, –0.05]

In both analyses, resilience accounted for a partial mediation: distress variables retained significant direct effects on well-being even after accounting for resilience. This pattern suggests that lower resilience partially transmits the negative association between distress and well-being, while also highlighting that distress exerts additional direct influence.

Interpretation:
Although cross-sectional data prevent causal claims, this exploratory analysis indicates that resilience plays a meaningful role in the pathway linking psychological distress to lower subjective well-being. This finding is consistent with theoretical models positioning resilience as a protective psychological mechanism and reinforces the manuscript's emphasis on positive-psychology constructs in post-disaster adjustment.

Supplementary Material Table S5: STROBE Checklist

A complete STROBE checklist for cross-sectional studies is provided below to document adherence to recommended reporting standards.

STROBE Checklist for Cross-Sectional Studies

Title and Abstract

1. (a) Indicate the study's design with a commonly used term in the title or abstract ✓
   (b) Provide an informative and balanced summary of what was done and found ✓

Introduction
2. Background/rationale — Explain the scientific background and rationale ✓
3. Objectives — State specific objectives or hypotheses ✓

Methods
4. Study design — Present key elements early in the paper ✓
5. Setting — Describe setting, locations, and dates ✓
6. Participants — Give eligibility criteria, sources and methods of selection ✓
7. Variables — Clearly define primary outcomes, predictors, confounders ✓
8. Data sources/measurement — Give details of methods of assessment ✓
9. Bias — Describe efforts to address bias ✓
10. Study size — Explain how sample size was determined ✓
11. Quantitative variables — Explain handling of quantitative variables ✓
12. Statistical methods — Describe all statistical methods, including:

- (a) All analyses ✓
- (b) Methods to examine subgroups ✓
- (c) How missing data were addressed ✓

- (d) How potential confounding was addressed ✓
- (e) Sensitivity analyses ✓

Results
13. Participants — Report numbers at each stage ✓
14. Descriptive data — Provide participant characteristics ✓
15. Outcome data — Report outcome events or summary measures ✓
16. Main results — Provide unadjusted and adjusted estimates ✓
17. Other analyses — Report subgroup and sensitivity analyses ✓

Discussion
18. Key results — Summarize main findings ✓
19. Limitations — Discuss limitations, potential biases ✓
20. Interpretation — Provide a cautious overall interpretation ✓
21. Generalizability — Discuss external validity ✓

Other Information
22. Funding — Give source of funding and role of funder ✓

Supplementary Material Table S6 : Participant-Flow Diagram

Total individuals approached: n = 753

|

├── Excluded during eligibility screening: n = 111

| • Current psychiatric diagnosis

| • Psychotropic medication use

| • Ongoing psychotherapy

| • Cognitive impairment or invalid/incomplete data

|

Final analytic sample: n = 642

|

└── Completed all psychological measures (BDI-II, BAI, PCL-5, WEMWBS, CD-RISC)
